# Modified control method of a motion compensated gangway

**DOI:** 10.1371/journal.pone.0351153

**Published:** 2026-07-10

**Authors:** Zhu Jiyue, Weitian Liu, Heng Yang

**Affiliations:** 1 School of Mechanical Engineering, Shaanxi University of Technology, Hanzhong, Shaanxi, China; 2 Aecc Aero Engine Control System Institute, Aero Engine Corporation of China, Wuxi, Jiangsu, China; 3 Tianjin FYTY Technology Co., Ltd, Tianjin, China; Tongji University, CHINA

## Abstract

With the growing demand for offshore operations such as wind farm maintenance and maritime transportation, motion-compensated gangways are widely employed on vessels to counteract ship motions in roll, pitch, and heave. However, the persistent and unpredictable ship motion disturbance, combined with nonlinearities in hydraulic system makes motion-compensated gangways more complex dynamic characteristics, which brings huge challenges for the controller design. To address the aforementioned problems, this paper proposes an improved cascade control strategy. Specifically, a dynamic model of the motion-compensated gangway, accounting for the ship motion disturbance, is established using Kane’s method, and the coupling between the gangway’s end-effector and ship motions is investigated. A multi-degree-of-freedom velocity compensation strategy is then introduced into the improved cascade control approach. Finally, simulations are included to validate the effectiveness of the proposed control strategy.

## Introduction

With the development of the marine economy, the rapid growth in demand for offshore wind farm installation and maintenance, offshore crane operations, and personnel transfer has presented higher requirements on operational capabilities [1–3]. The equipment for the aforementioned offshore operation will inevitably experience performance deterioration due to the persistent vertical motions [4]. To address the vessel motions, motion-compensated gangways have been utilized as a current solution. Nevertheless, considering the complex dynamic characteristics and the persistent disturbances from the vessel motions, the stabilization technique for the motion compensated gangway still requires further investigation [5,6].

The motion-compensated gangway is a ship-mounted mechanical system, and numerous research results have been reported over the past two decades. Regarding single-degree-of-freedom heave compensation systems, Kuchler et al. addressed the problem of motion sensor latency by utilizing a feedback linearization controller based on short-term prediction of ship motion, and satisfactory experimental results were obtained [7]. Chen et al. proposed a novel adaptive robust control strategy combining an Equivalent Model Predictive Control (EMPC) method with a Bias Proportional-Integral-Derivative (BPID) framework, and the undesired effect of ship heave motion on the suspended payload was effectively improved [8]. Other methods, e.g., the double closed-loop PID control [9], fuzzy adaptive PID control [10], neural network PID control [11] and deep learning-based adaptive methods [12] etc. were also reported. As for offshore cranes, various control methods have been proposed by many scholars to address the ship motion disturbance and system parameter uncertainties. The adaptive control scheme [13] and neural network control scheme [14] have been developed, achieved asymptotically convergent results and improved the control accuracy of cargo transfer. Regarding motion-compensated gangways, Qiu designed an offshore hybrid gangway and established its dynamic model using the Newton-Euler method [15]. Li investigated the real-time motion of the gangway with ship hydrodynamic forces considered. Calculation results indicated that when the significant wave height reached 3.25m, the vertical motion between the gangway departure and the landing points could reach up to 10 m [16]. Yu et al. designed a motion-compensated gangway employing a typical PID control strategy [17]. Liang et al. proposed an improved controller based on inverse dynamic; simulation results presented satisfactory control performance. Regrettably, the dynamic characteristic of the actuator system was not considered [18]. Zhao et al. proposed a vision-guided docking system for a novel series-parallel hybrid gangway system, introduced a double closed-loop PID control method to compensate for the hull motion, and validated its effectiveness through experiments. However, this series-parallel hybrid gangway system had a complicated mechanical structure and had not been utilized in engineering applications [19]. Wang et al. proposed three compensation methods to compensate for roll, pitch, and heave motions, respectively, and then designed a fuzzy PID controller with feedforward compensation for the turbine access system (TAS), achieving satisfactory control performance [20]. However, the motion of a ship constitutes a multi-degree-of-freedom spatial motion, thus the methods are limited in use. Wang et al. made an in-depth investigation on the Ampelmann system. An adaptive super-twisting global fast terminal sliding-mode controller with velocity feed-forward was designed for the parallel mechanism, and a dynamic feed-forward with the PD controller was utilized for the serial mechanism. Simulation results indicate that the proposed approach achieves significant improvements in trajectory tracking and disturbance suppression [21]. Tan et al. combined PID, proximal policy optimization, and multi-agent reinforcement learning for the three-DOF wave compensation control of the ship-mounted Stewart platform. The effectiveness of the proposed method was validated through simulation and experimentation, achieving a compensation efficiency exceeding 94% [22]. For the above two methods, the controller require extensive computations and make numerous decisions, therefore imposing stringent demands on the hardware.

From the above literature review, it can be observed that most studies do not sufficiently investigate the coupling mechanism between the ship and the mechanical system. The dynamic models fail to sufficiently reflect the ship-induced motion disturbance on the gangway. Some studies do not consider the effect of the dynamic characteristics of the actuator system on wave compensation performance. To address these problems, this paper investigates a stabilization strategy for the motion-compensated gangway. Specifically, a dynamic model of the motion-compensated gangway system with the ship motion disturbance considered is established. Then, the motion coupling mechanism between the ship motion and the gangway’s end-effector is analyzed. Finally, a modified control method based on velocity feedforward is introduced, and simulation results are provided to demonstrate the effectiveness of the proposed control strategy.

The contributions and novelties of this paper reside in the following aspects:

(1) Different from the aforementioned study in [22], this paper fully considers the dynamic characteristics of the actuator system in the motion-compensated gangway.
(2) Unlike the existing ideas, this work first analyzes the motion coupling mechanism between the motion-compensated gangway system and the ship.
(3) An improved controller based on a velocity feedforward compensator is proposed.

The remaining parts of this paper are organized as follows. In the Problem formulation section, the system dynamic model of the motion-compensated gangway is set up. The Design the improved controller section analyzes the motion coupling mechanism between the motion-compensated gangway system and the ship, and designs an improved controller based on the velocity feedforward compensator. The Simulation results section provides some simulation results to illustrate performance of the controller, and the Conclusion section gives the conclusions of this paper.

**Notations**. Throughout the paper, **S**_α_ and **C**_α_ are utilized to denote functions **sin**α and **cos**α, respectively; **S**(***v***) denotes a skew symmetric matrix of vector ***v***, diag(***k***) is a corresponding diagonal matrix of a vector ***k***.

## Problem formulation

### Nonlinear model of the motion-compensated gangway system

**Fig 1:**
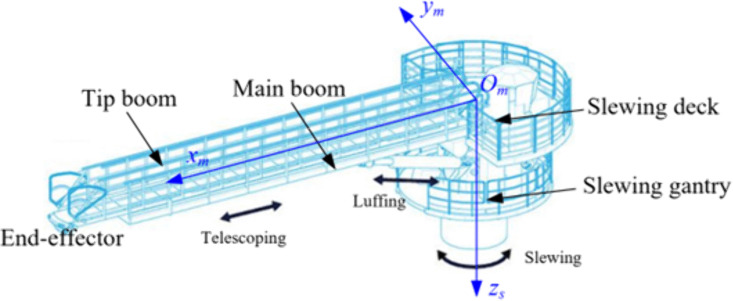
Three-degree-of-freedom serial robot [23]. This figure is a recreated version for illustrative purposes only. It is not identical to the original image and complies with the CC BY 4.0 license.

As shown in Fig 1, the motion-compensated gangway is primarily utilized for personnel transfer and cargo transportation; i.e., a three-degree-of-freedom serial robot is mounted (containing the slewing, luffing, and telescoping joints) on the vessel to maintain the robot’s end-effector motionless relative to the inertial frame. The ship motions are continuously measured by a motion sensor, e.g., a motion reference unit (MRU), and the robot’s joints are then controlled to compensate for these vessel motions.

**Fig 2:**
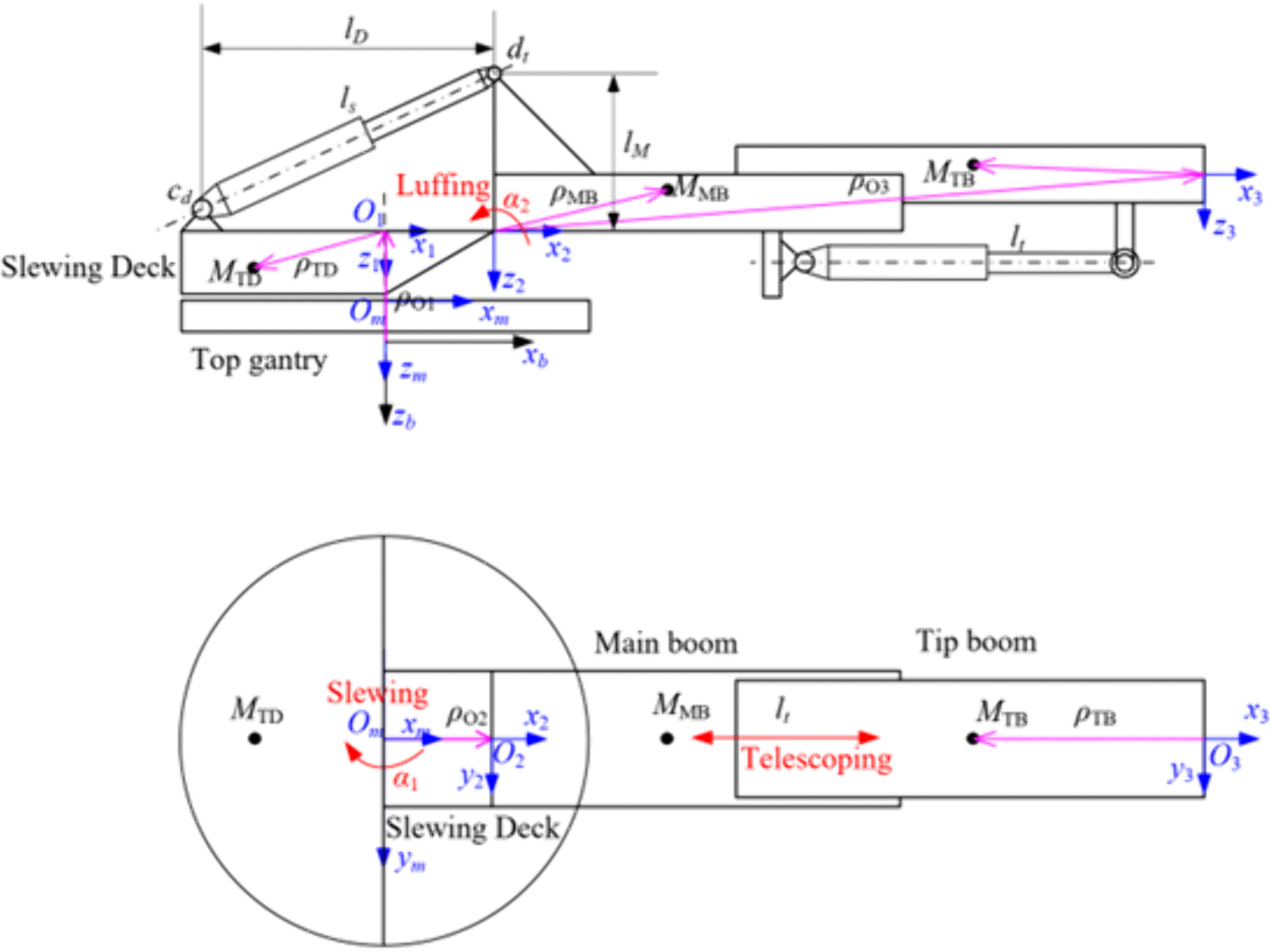
Illustration for motion compensated gangway.

Before establishing the dynamic model of the ship-mounted robot, we define the ship-fixed coordinate system ***O***_***m***_-*X*_***m***_***Y***_***m***_***Z***_***m***_, where the origin ***O***_***m***_ is located at the installation point of the motion sensor. In this frame, the ***X***_***m***_***-axis*** points to the forward moving direction of the ship, the ***Z***_***m***_***-axis*** is perpendicular to the ship deck, and the ***Y***_***m***_***-axis*** is determined by the right-hand rule. The inertial coordinate system ***O***_***b***_***-X***_***b***_***Y***_***b***_***Z***_***b***_ is fixed to the earth, with the ***X***_***m***_***-axis*** parallel to the ***X***_***b***_***-axis*** and the ***Z***_***m***_***-axis*** initially parallel to the ***Z***_***b***_***-axis*** at the initial state. As shown in Fig 2, a body-fixed coordinate system ***O***_***i***_***-X***_***i***_***Y***_***i***_***Z***_***i***_ ***(i = 1 − 3)*** is established at each joint, where the direction of the ***X***_***i***_***-axis*** aligns with ***X***_***m***_, and ***Z***_***m***_ remains perpendicular to the ship deck. Among these coordinate systems, the ***O***_***3***_ coordinate system represents the end-effector of the motion-compensated gangway.

The vector ***ρ***_***o*1**_ denotes the displacement of the center of the slewing joint in the ***O***_***m***_ frame, the vector ***ρ***_***o***2_ denotes the displacement of the center of the luffing joint relative to the ***O***_**1**_ coordinate system, and the vector ***ρ***_***o***3_ represents the displacement of the center of the robot end-effector in the ***O***_***2***_ coordinate system. It should be noted that the vector ***ρ***_***o***3_ includes the telescoping joint variable *d***.**

To establish the dynamic model of the ship-mounted robot, the pose of the ship should be described first; this paper considers the three-degree-of-freedom motions of the ship (roll, pitch, and heave). The orientation of frame ***O_m_*** relative to frame ***O_b_*** can be described by the following Euler transformation:


$$\begin{array}{c} R_{bm}=\left[\begin{array}{ccc} @ccc@\text{c}\theta_{m} & \text{s}\theta_{m}\cdot \text{s}\varphi_{m} & \text{s}\theta_{m}\cdot \text{c}\varphi_{m}\\ 0 & \text{c}\varphi_{m} & -\text{s}\varphi_{m}\\ -\text{s}\theta_{m} & \text{c}\theta_{m}\cdot \text{s}\varphi_{m} & \text{c}\theta_{m}\cdot \text{c}\varphi_{m} \end{array}\right] \end{array}$$ (1)

For the translational position of the ship relative to the inertial frame, it can be described by the displacement vector ***t***_***m***_ **= [0; 0; *z***_**m**_**].**

The rotation transformation matrices for the two rotational joints of the robot can be described as follows:


$$\begin{array}{c} R_{\text{slewing}}=R_{z}\left(\alpha_{\text{1}}\right) \end{array}$$ (2)

$$\begin{array}{c} R_{\text{luffing}}=R_{y}\left(\alpha_{\text{2}}\right) \end{array}$$ (3)

For the convenience of subsequent analysis, the ship motion is defined as the generalized displacement ***q***_***m***_ **= [*θ***_***m***_, φ_***m***_, ***z***_***m***_], and the three joint displacements of the robot are defined as ***q***_***t***_ = [*α*_***1***_, ***α***_***2***_, ***l***_***t***_]. The entire generalized displacement is denoted as ***q =* [*q***_***m***_***, q***_***t***_]. The displacement of the robot end-effector in the inertial frame is given as follows:


$$\begin{array}{c} r_{o3}=t_{m}+R_{bm}\rho_{o1}+R_{bm}R_{\text{slewing}}\rho_{o2}+R_{bm}R_{\text{slewing}}R_{\text{luffing}}\rho_{o3} \end{array}$$ (4)

We now proceed to the velocity analysis of the subsystems. First, the angular velocity equation of the ship motion in the inertial frame is given as follows:


$$\begin{array}{c} \omega_{m}=\left[\begin{array}{ccc} @ccc@0 & 1 & 0\\ c\varphi_{m} & 0 & 0\\ s\varphi_{m} & 0 & 0 \end{array}\right]=J_{\omega m}{\dot{q}}_{m} \end{array}$$ (5)

**Fig 3:**
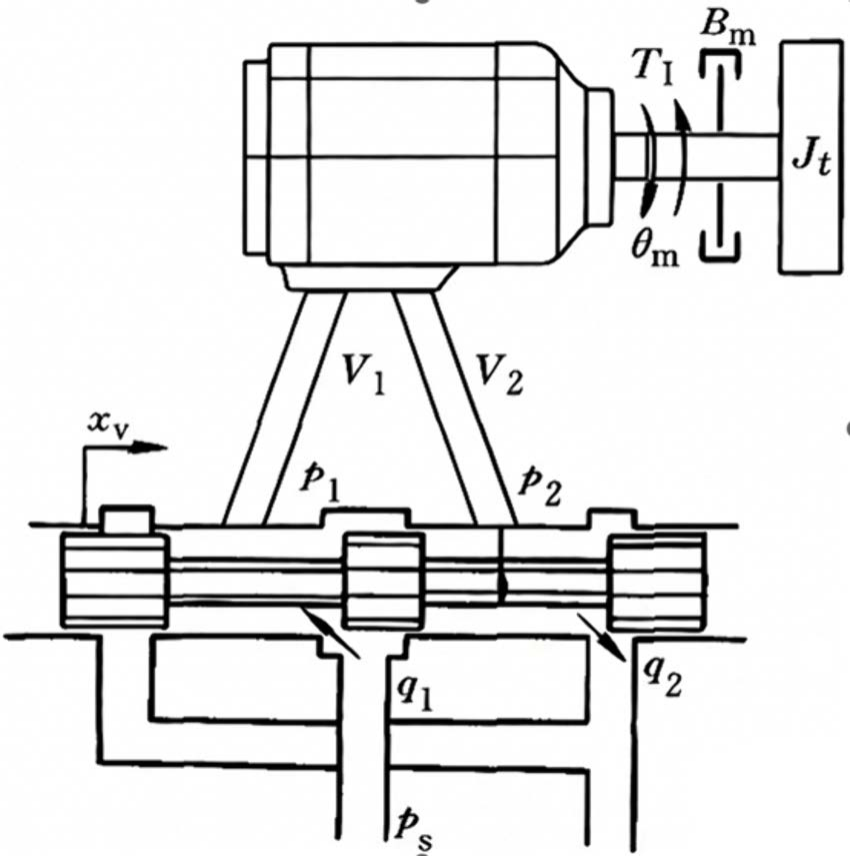
Schematic diagram of the valve-controlled hydraulic motor. This figure is a recreated version for illustrative purposes only. It is not identical to the original image and complies with the CC BY 4.0 license.


Using the superposition principle of angular velocity, the angular velocity of the slewing deck (in Fig 3) in the ***O***_***m***_ frame can be expressed as follows:

$$\begin{array}{c} \omega_{TD}=\omega_{m}+\omega_{Tm}=\left[\begin{array}{cc} @cc@\overset{\text{T}}{\underset{bm}{R}}J_{\omega m}, & J_{Tm} \end{array}\right]\dot{q}=J_{\omega TD}\dot{q} \end{array}$$ (6)

where ***J*_*TD*_=[0,0,0;0,0,0;1,0,0]**.

Considering the ship’s heave motion, the velocity of point ***O***_***1***_ on the slewing deck is given by:


$$\begin{array}{c} v_{o1}={\dot{t}}_{m}+\omega_{o1}\times \rho_{o1}=\left[\begin{array}{cc} @cc@\left(\overset{\text{T}}{\underset{bm}{R}}J_{tm}-S\left(\rho_{o1}\right)\overset{\text{T}}{\underset{bm}{R}}J_{\omega m}\right) & {\text{0}}_{3\times 3} \end{array}\right]\dot{q}=J_{o1}\dot{q} \end{array}$$ (7)

where ***J***_***tm***_ = **[0,0,0;0,0,0;0,0,1]**, and ***0***_***3×3***_ denotes a 3×3 zero matrix.

The velocity of the centroid of the slewing deck can be further calculated as follows:


$$\begin{array}{c} v_{TD}=v_{o1}+\omega_{TD}\times \rho_{o1}=\left(J_{o1}-S\left(\rho_{o1}\right)J_{\omega TD}\right)\dot{q}=J_{TD}\dot{q} \end{array}$$ (8)

The velocity of point O₂ on the slewing deck is given by:


$$\begin{array}{c} v_{TD}=v_{o1}+\omega_{TD}\times \rho_{o1}=\left(J_{o1}-S\left(R_{\text{slewing}}\rho_{o2}\right)J_{\omega TD}\right)\dot{q}=J_{TD}\dot{q} \end{array}$$ (9)

Similarly, the angular velocity of the main boom in the ship-fixed coordinate system is expressed as:


$$\begin{array}{c} \omega_{MB}=\omega_{TD}+J_{\omega MT}{\dot{q}}_{t}=\left(J_{\omega TD}+\left[\begin{array}{cc} @cc@{\text{0}}_{3\times 3} & J_{\omega MT} \end{array}\right]\right)\dot{q}=J_{\omega MB}\dot{q} \end{array}$$ (10)

where ***J*_*ωMT*_ = [0,0,0;0,1,0;0,0,0]**.

The centroid velocity of the main boom in the ship-fixed coordinate system is:


$$\begin{array}{c} v_{MB}=v_{o2}+\omega_{MB}\times R_{\text{slewing}}\rho_{MB}=\left(J_{o2}-S\left(R_{\text{slewing}}\rho_{MB}\right)J_{\omega MB}\right)\dot{q}=J_{MB}\dot{q} \end{array}$$ (11)

The angular velocity of the gangway boom in the ship-fixed coordinate system is equivalent to that of the main boom, i.e., *ω*_*TB*_ = *ω*_*MB*_.

The velocity of point ***O***₃ on the gangway boom in the ship-fixed coordinate system is given by:


$$\begin{array}{c} \begin{array}{c} v_{o3}=v_{o2}+\omega_{TB}\times R_{\text{slewing}}R_{\text{luffing}}\rho_{o3}+R_{\text{slewing}}R_{\text{luffing}}{\dot{\rho }}_{o3}=\\ \left(J_{o2}-S\left(R_{\text{slewing}}R_{\text{luffing}}\rho_{o3}\right)J_{\omega MB}+\left[\begin{array}{cc} @cc@{\text{0}}_{3\times 3} & R_{\text{slewing}}R_{\text{luffing}}J_{d} \end{array}\right]\right)\dot{q}=J_{o3}\dot{q} \end{array} \end{array}$$ (12)

where ***J*_*d*_ = [0,0,0;0,1,0;0,0,0].**

The velocity of the centroid on the gangway boom in the ship-fixed coordinate system is given by:


$$\begin{array}{c} v_{TB}=v_{o3}+\omega_{TB}\times R_{\text{slewing}}R_{\text{luffing}}\rho_{TB}=\left(J_{o3}-S\left(R_{\text{slewing}}R_{\text{luffing}}\rho_{TB}\right)J_{\omega MB}\right)\dot{q}=J_{TB}\dot{q} \end{array}$$ (13)

Through the aforementioned kinematic analysis, it can be seen that the velocity Jacobian matrices consist of two parts: one induced by the ship motion, and the other induced by the robot joint motion, e.g., ***J***_*TD*_=[***J***_*TDm*_,***J***_*TDt*_], where ***J***_*TDm*_ corresponds to the ship motion components and ***J***_*TDt*_ corresponds to the robot joint motion components. Therefore, they have a unified form of [***J***_*m*_,***J***_*t*_].

We now proceed to the dynamic modeling using the Kane’s method. Firstly, the Jacobian matrices are utilized as projection operators to project the inertial forces, the inertial moments, the gravitational forces, and the joint actuator forces into the task space as generalized inertia forces and generalized actuator forces. Then, the dynamic equations can be obtained based on D’Alembert Theory.

For the slewing deck, the generalized inertia force can be written as follows:


$$\begin{array}{c} \begin{array}{c} \overset{I}{\underset{TD}{F}}=-\overset{\text{T}}{\underset{TDt}{J}}m_{TD}\left(J_{TDt}{\ddot{q}}_{t}+{\dot{J}}_{TDt}{\dot{q}}_{t}+J_{TDm}{\ddot{q}}_{m}+{\dot{J}}_{TDm}{\dot{q}}_{m}\right)-\\ \overset{\text{T}}{\underset{\omega TDt}{J}}\left(I_{TD}\left(J_{\omega TDt}{\ddot{q}}_{t}+{\dot{J}}_{\omega TDt}{\dot{q}}_{t}+J_{\omega TDm}{\ddot{q}}_{m}+{\dot{J}}_{\omega TDm}{\dot{q}}_{m}\right)+S\left(\omega_{TD}\right)I_{TD}\left(J_{\omega TDt}{\dot{q}}_{t}+J_{\omega TDm}{\dot{q}}_{m}\right)\right) \end{array} \end{array}$$ (14)

where *m*_*TD*_ is the mass of the slewing deck, and ***I***_TD_ denotes the inertia tensor of the slewing deck in the inertial frame, with ***I***_*TD*_ = ***R***_bm_***R***_slewing_***I***_*TDC*_(***R***_bm_***R***_slewing_)^T^. Herein, ***I***_*TDC*_ denotes the inertia tensor of the slewing deck about its centroid.

For the main boom of the robot, the generalized inertia force can be expressed as follows:


$$\begin{array}{c} \begin{array}{c} \overset{I}{\underset{MB}{F}}=-\overset{\text{T}}{\underset{MBt}{J}}m_{MB}\left(J_{MBt}{\ddot{q}}_{t}+{\dot{J}}_{MBt}{\dot{q}}_{t}+J_{MBm}{\ddot{q}}_{m}+{\dot{J}}_{MBm}{\dot{q}}_{m}\right)-\\ \overset{\text{T}}{\underset{\omega MBt}{J}}\left(I_{MB}\left(J_{\omega MBt}{\ddot{q}}_{t}+{\dot{J}}_{\omega MBt}{\dot{q}}_{t}+J_{\omega MBm}{\ddot{q}}_{m}+{\dot{J}}_{\omega MBm}{\dot{q}}_{m}\right)+S\left(\omega_{MB}\right)I_{MB}\left(J_{\omega MBt}{\dot{q}}_{t}+J_{\omega MBm}{\dot{q}}_{m}\right)\right) \end{array} \end{array}$$ (15)

where *m*_*MB*_ is the mass of the main boom, and ***I***_*MB*_ represents the inertia tensor of the main boom in the inertial frame, with ***I***_*MB*_ = ***R***_*bm*_***R***_*s*lewing_***R***_luffing_***I***_*MBC*_(***R***_*bm*_***R***_slewing_***R***_luffing_)^*T*^. Herein, ***I***_*MBC*_ denotes the inertia tensor of the main boom about its centroid.

For the gangway boom, the generalized inertia force can be expressed as follows:


$$\begin{array}{c} \begin{array}{c} \overset{I}{\underset{TB}{F}}=-\overset{\text{T}}{\underset{TBt}{J}}m_{TB}\left(J_{TBt}{\ddot{q}}_{t}+{\dot{J}}_{TBt}{\dot{q}}_{t}+J_{TBm}{\ddot{q}}_{m}+{\dot{J}}_{TBm}{\dot{q}}_{m}\right)-\\ \overset{\text{T}}{\underset{\omega TBt}{J}}\left(I_{TB}\left(J_{\omega TBt}{\ddot{q}}_{t}+{\dot{J}}_{\omega TBt}{\dot{q}}_{t}+J_{\omega TBm}{\ddot{q}}_{m}+{\dot{J}}_{\omega TBm}{\dot{q}}_{m}\right)+S\left(\omega_{TB}\right)I_{TB}\left(J_{\omega TBt}{\dot{q}}_{t}+J_{\omega TBm}{\dot{q}}_{m}\right)\right) \end{array} \end{array}$$ (16)

where *m*_*TB*_ is the mass of the gangway boom, and ***I***_*TB*_ represents the inertia tensor of the gangway boom in the inertial frame, with ***I***_*TB*_ = ***R***_*bm*_***R***_slewing_***R***_luffing_*I*_*TBC*_(***R***_*bm*_***R***_slewing_***R***_luffing_)^T^. Here, ***I***_*TBC*_ denotes the inertia tensor of the gangway boom about its centroid.

Finally, the generalized actuator force is given by:


$$\begin{array}{c} F_{pa}=\overset{\text{T}}{\underset{TBt}{J}}m_{TB}g+\overset{\text{T}}{\underset{MBt}{J}}m_{MB}g+\overset{\text{T}}{\underset{TBt}{J}}m_{TB}g+\tau \end{array}$$ (17)

where **g** is the gravity vector, and **τ** = [τ₁, τ₂, τ₃]ᵀ represents the actuator forces and torques of each joint.

After complex derivation and mathematical computation, the dynamic equation of the motion compensated gangway can be formulated as follows:


$$\begin{array}{c} M_{t}{\ddot{q}}_{t}+C_{t}{\dot{q}}_{t}-G_{t}+M_{m}{\ddot{q}}_{m}+C_{m}{\dot{q}}_{m}=\tau \end{array}$$ (18)

With


$$\begin{array}{c} M_{t}=\overset{\text{T}}{\underset{TBt}{J}}m_{TB}J_{TBt}+\overset{\text{T}}{\underset{\omega TBt}{J}}I_{TB}J_{\omega TBt} \end{array}$$ (19)

$$\begin{array}{c} C_{t}=\overset{\text{T}}{\underset{TBt}{J}}m_{TB}{\dot{J}}_{TBt}+\overset{\text{T}}{\underset{\omega TBt}{J}}I_{TB}{\dot{J}}_{\omega TBt}+\overset{\text{T}}{\underset{\omega TBt}{J}}I_{TB}S\left(\omega_{TB}\right)I_{TB}J_{\omega TBt} \end{array}$$ (20)

$$\begin{array}{c} G_{t}=\overset{\text{T}}{\underset{TBt}{J}}m_{TB}g+\overset{\text{T}}{\underset{MBt}{J}}m_{MB}g+\overset{\text{T}}{\underset{TBt}{J}}m_{TB}g \end{array}$$ (21)

$$\begin{array}{c} M_{m}=\overset{\text{T}}{\underset{TBt}{J}}m_{TB}J_{TBm}+\overset{\text{T}}{\underset{\omega TBt}{J}}I_{TB}J_{\omega TBm} \end{array}$$ (22)

$$\begin{array}{c} C_{m}=\overset{\text{T}}{\underset{TBt}{J}}m_{TB}{\dot{J}}_{TBm}+\overset{\text{T}}{\underset{\omega TBt}{J}}I_{TB}{\dot{J}}_{\omega TBm}+\overset{\text{T}}{\underset{\omega TBt}{J}}I_{TB}S\left(\omega_{TB}\right)I_{TB}J_{\omega TBm} \end{array}$$ (23)

Considering that the luffing joint of the motion-compensated gangway is actuated by a hydraulic cylinder, Eq. (18) can be expressed as:


$$\begin{array}{c} M_{t}{\ddot{q}}_{t}+C_{t}{\dot{q}}_{t}-G_{l}+M_{m}{\ddot{q}}_{m}+C_{m}{\dot{q}}_{m}=J_{T}\tau_{a} \end{array}$$ (24)

where $J_{T}=\left[\begin{array}{ccc} @ccc@\text{ratio} & 0 & 0\\ 0 & \frac{L_{M}\cdot L_{D}\cdot cos\left(\alpha_{\text{2}}\right)}{l_{s}} & 0\\ 0 & 0 & 1 \end{array}\right]$, $\tau_{a}=[\tau_{\text{1}},f_{l},f_{t}{]}^{T}$ represents the actuator forces of each joint.

Here, ratio denotes the gear reduction ratio of the slewing joint, *l*_*D*_ and *l*_*M*_ represent the positions of the two hinge points of the hydraulic cylinder, *f*_*l*_ is the actuator force of the luffing joint, and *f*_*t*_ is the actuator force of the telescoping joint.

### Actuator subsystem model

In the motion-compensated gangway system, the electrohydraulic motor (for the slewing joint) and actuators (for the luffing and telescoping joints) are chosen for the active control of the gangway. The actuator subsystem model for the slewing joint can be described as the valve-controlled motor in Fig 3.

In the above schematic diagram, *J*_*t*_ represents the moment of inertia of the motor shaft, *B*_*m*_ is the rotational damping, *T*_*I*_ denotes the motor torque, *θ*_*m*_ is the angular displacement of the motor, *p*_1_ and *p*_2_ are the pressures in the two chambers of the motor, *q*_1_ and *q*_2_ are the flow rates through the two chambers, *V*_1_ and *V*_2_ are the control volumes of the two chambers, *x*_*v*_ is the spool displacement of the servo valve, *p*_*s*_ is the supply pressure, and *p*_0_ ≈ 0 is the return pressure.

**Fig 4:**
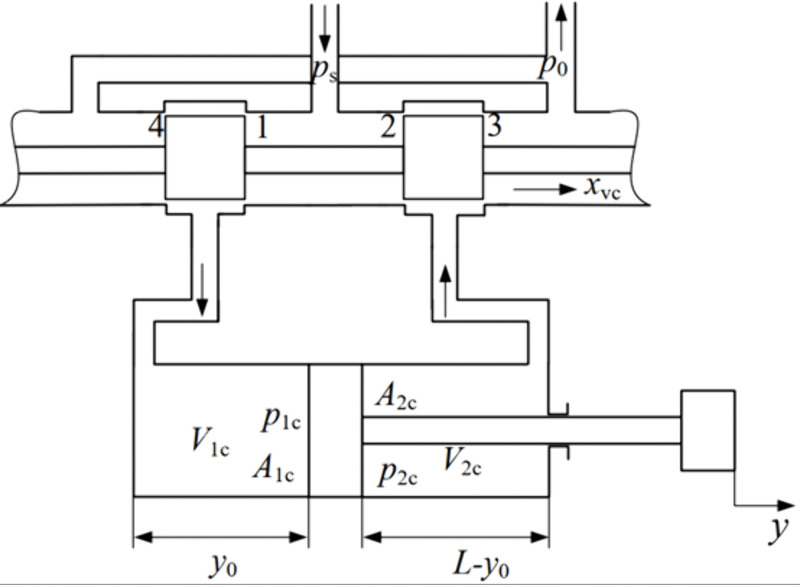
Schematic diagram of the valve-controlled hydraulic cylinder. This figure is a recreated version for illustrative purposes only. It is not identical to the original image and complies with the CC BY 4.0 license.

For the luffing and telescoping joints, we can utilize the valve-controlled cylinder model to describe the dynamic process, whose schematic diagram is shown in Fig 4. In the diagram, the sub-indexes 1 and 2 refer, respectively, to the rodless chamber and the rod chamber of a piston; *V*_1c_ = *V*_1c0_ + *A*_1c_*y* and *V*_2c_ = *V*_2c0_ - *A*_2c_*y* represent the control volumes of the chambers, where *V*_1c0_ and *V*_2c0_ is the initial volumes, *A*_1c_ and *A*_2c_ denote the piston areas of the respective chambers, *p*_1c_ and *p*_2c_ are the chamber pressures, and *y* denotes the displacement of the hydraulic cylinder, and *x*_*vc*_ is the spool displacement of the servo valve. The detailed simulation model can be referred to in [24].

Since the frequency bandwidth of the wave spectrum is much lower than that of the servo valve, the dynamic of the servo valve are neglected here. To facilitate controller design, the simulation model in this paper is simplified into the following form:


$$\begin{array}{c} {\dot{p}}_{L}\text{=}K_{\text{0}}f\left({\overset{―}{p}}_{L}\right)u-D_{t}{\dot{q}}_{t}-C_{tc}p_{L} \end{array}$$ (25)

where *p*_*L*_ represents the load pressure of the hydraulic system, *K*_0_ is a constant and determined by the rated flow rate of servo valve ***Q***_*N*_ and the parameter ***D***_*t*_, ***f***(*p*_*L*_) is a diagonal matrix of nonlinear functions representing the hydraulic system nonlinearities and for each joint it has a form of $f_{i}\left({\overset{―}{p}}_{L}\right)=\left\{ \begin{array}{c} @l@\sqrt{1-{\overset{―}{p}}_{L}},u\geq 0\\ \sqrt{n_{\text{0}}+{\overset{―}{p}}_{L}},u<0 \end{array}\right.$, *n*_0_ denotes the ratio of area gradients of the spool ports, ${\overset{―}{p}}_{L}$  = *p*_*L*_/*p*_*s*_, and *p*_*L*_ is the load pressure, *u* is the input current to the servo valve, ***D***_*t*_=[*D*_*m*_, *A*_1_, *A*_*t*_], *D*_*m*_ is the displacement of the hydraulic motor, *A*_1_ is the ram area of the rodless chamber of hydraulic cylinder for the luffing joint, *A*_*t*_ is the ram area of the rodless chamber of the hydraulic cylinder for the telescoping joint, and *C*_*tc*_ is the total leakage coefficient.

The state variables are defined as ***x*** = [*x*₁, *x*₂, *x*₃] = [*α*₁, *l_s_*, *l_t_*]. The entire control system is consequently transformed into the following state-space form:


$$\begin{array}{c} {\dot{x}}_{\text{1}}=x_{\text{2}} \end{array}$$ (26)

$$\begin{array}{c} M_{t}{\dot{x}}_{\text{2}}\text{=}D_{t}x_{\text{3}}-C_{tc}x_{\text{2}}+G_{t}-M_{m}{\ddot{q}}_{m}-C_{m}{\dot{q}}_{m} \end{array}$$ (27)

$$\begin{array}{c} {\dot{x}}_{\text{3}}\text{=}K_{\text{0}}f\left({\overset{―}{p}}_{L}\right)u-D_{t}x_{\text{2}}-C_{tc}x_{\text{3}} \end{array}$$ (28)

## Design the improved controller

### Multi-degree-of-freedom velocity compensator

The motion-compensated gangway is designed to accomplish two primary tasks: first, to maintain the end-effector position motionless in the inertial frame; second, to complete docking with the offshore platform and impose a constant contact force on the platform. The first task is the foundation for the second. Therefore, the system model for the first task is analyzed herein. This section focuses on analyzing the motion coupling mechanism between the ship and the end-effector. For this purpose, Eq. (24) is transformed into the dynamic equation in the task space, which takes the following form:


$$\begin{array}{c} M_{o}{\dot{v}}_{o3}+C_{o}v_{o3}-G_{o}+M_{om}{\ddot{q}}_{m}+C_{om}{\dot{q}}_{m}=\tau_{m} \end{array}$$ (29)

The aforementioned model is only suitable for theoretical analysis. Therefore, the specific expressions for ***M***_*o*_, ***C***_*o*_, ***G***_*o*_, ***M***_*om*_, and ***C***_*om*_ are not provided here. This formulation also describes the linearized model of the gangway system at the docking position. Although this model cannot fully and precisely describe the dynamic characteristics of the motion compensated gangway, it can provide some inspiration for controller design. Considering the low frequency of vessel motions, the velocity term in Eq. (29) is neglected, and the main effect of the gravity term is the steady-state error of the control system and this effect is thus set aside during the subsequent analysis. To facilitate qualitative analysis, the linearized model from Eq. (25) is used.


$$\begin{array}{c} {\dot{p}}_{L}=\frac{4\beta_{e}}{V_{t}}\left(k_{q}u-D_{t}{\dot{q}}_{t}-k_{ce}p_{L}\right) \end{array}$$ (30)

where *β*_*e*_ is the bulk modulus of the hydraulic fluid, *k*_*q*_ is the flow coefficient of the servo valve, *u* is the input current to the servo valve, *k*_*ce*_ is the total leakage coefficient, and *V*_*t*_ is the control volume.

**Fig 5:**
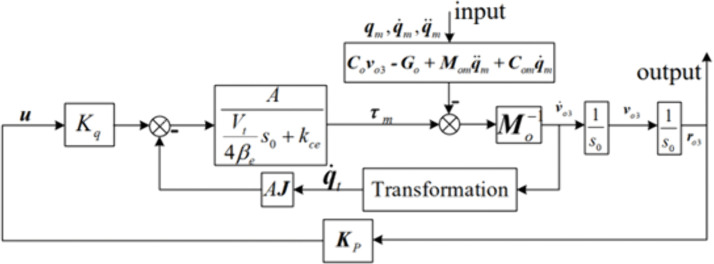
Control system block diagram.

To evaluate the control system performance, a proportional controller is used for the displacement feedback analysis. The system block diagram is shown in Fig 5.

Further, by taking the ship motion as the input and the end-position of the motion-compensated gangway as the output, and combining Equations (29) and (30) with Laplace transformation, the input-output frequency characteristics of the control system are obtained as:


$$\begin{array}{c} \left(\left(\frac{V_{t}}{4\beta_{e}}s_{\text{0}}+k_{ce}\right)M_{o}\overset{\text{2}}{\underset{\text{0}}{s}}+A^{\text{2}}JT^{-1}s_{\text{0}}\right)q=Ak_{q}u+A^{\text{2}}JT^{-1}s_{\text{0}}q_{m}-\left(\frac{V_{t}}{4\beta_{e}}s_{\text{0}}+k_{ce}\right)M_{o}\overset{\text{2}}{\underset{\text{0}}{s}}q_{m} \end{array}$$ (31)

where *s*_0_ is the Laplace operator. If a proportional gain *u*=−*K*_*P*_*q* is applied, Equation (31) can be rewritten as:


$$\begin{array}{c} \left(\left(\frac{V_{t}}{4\beta_{e}}s_{\text{0}}+k_{ce}\right)M_{o}\overset{\text{2}}{\underset{\text{0}}{s}}+A^{\text{2}}JT^{-1}s_{\text{0}}+Ak_{q}K_{P}\right)q=A^{\text{2}}JT^{-1}s_{\text{0}}q_{m}-\left(\frac{V_{t}}{4\beta_{e}}s_{\text{0}}+k_{ce}\right)M_{b}\overset{\text{2}}{\underset{\text{0}}{s}}q_{m} \end{array}$$ (32)

Eq. (27) describes the dynamic relationship between ship motion and the end-effector response of the motion-compensated gangway, and indicates that the velocity of ship and acceleration act as inputs to the motion response of the gangway end-effector. It should be noted that the ship motions induced by ocean waves are characterized by low frequency range. Under such conditions, the term the term *k_ce_ + s_0_V_t_ / 4β_e_* in Eq. (32) approaches zero and can be neglected, with ship motion velocity being the dominant factor. Eq. (32) indicates the velocity coupling mechanism between the gangway’s end-effector and ship motion. Introducing the ship motion velocity as a feedforward component in the controller design can, to some extent, enhance motion compensation performance.

In the actual system, the nonlinearities in the actuator subsystem should also be considered. When the gangway performs wave compensation, its end-effector remains nearly stationary relative to the inertial frame. The first term in Eq. (32) essentially represents the induced flow rate due to the ship motion. By setting ***v****_o_*_3_ = 0, the velocity vector required for the slewing, luffing, and telescoping joints can be solved as follows:


$$\begin{array}{c} {\dot{q}}_{tm}={\left(R_{\text{slewing}}R_{\text{luffing}}J_{d}\right)}^{-1}\left(J_{o2}-S\left(R_{\text{slewing}}R_{\text{luffing}}\rho_{o3}\right)J_{\omega MB}\right){\dot{q}}_{m} \end{array}$$ (33)

Further considering the flow nonlinearity of the servo valve in the hydraulic system, the velocity compensator takes the following form:


$$\begin{array}{c} u_{m}=D_{t}{\left(K_{\text{0}}f\left({\overset{―}{p}}_{L}\right)\right)}^{-1}{\dot{q}}_{tm} \end{array}$$ (34)

### Design the motion controller

To ensure that the end-effector of the motion-compensated gangway remains motionless relative to the inertial frame, and since the gangway’s end-position cannot be directly measured, we must control the joint displacements to indirectly regulate the end-effector position. As indicated by Eq. (4), a trajectory generation module of the motion compensated gangway can compute the joint displacements based on the ship’s motion pose and the desired end-effector pose and the results are as follows:


$$\begin{array}{c} r_{d}=\overset{\text{T}}{\underset{bm}{R}}\left(r_{o3d}-t_{m}\right) \end{array}$$ (35)

$$\begin{array}{c} \alpha_{1d}\text{=}arctan\left(r_{d}\left(2\right)/r_{d}\left(1\right)\right)-arcsin\left(\frac{\rho_{o2}\left(2\right)+\rho_{o30}\left(2\right)}{\sqrt{r_{d}{\left(1\right)}^{\text{2}}+r_{d}{\left(2\right)}^{\text{2}}}}\right) \end{array}$$ (36)

$$\begin{array}{c} \begin{array}{c} \alpha_{2d}\text{=}arctan\left(\frac{\rho_{o1}\left(3\right)+\rho_{o2}\left(3\right)-r_{d}\left(3\right)}{r_{d}\left(1\right)c_{\alpha_{\text{1}}}+r_{d}\left(2\right)s_{\alpha_{\text{1}}}-\rho_{o2}\left(1\right)}\right)-\\ arcsin\left(\frac{-\rho_{o30}\left(3\right)}{\sqrt{{\left(\rho_{o1}\left(3\right)+\rho_{o2}\left(3\right)-r_{d}\left(3\right)\right)}^{\text{2}}+{\left(r_{d}\left(1\right)c_{\alpha_{\text{1}}}+r_{d}\left(2\right)s_{\alpha_{\text{1}}}-\rho_{o2}\left(1\right)\right)}^{\text{2}}}}\right) \end{array} \end{array}$$ (37)

$$\begin{array}{c} l_{sd}\text{=}\sqrt{\overset{\text{2}}{\underset{D}{l}}+\overset{\text{2}}{\underset{M}{l}}-2l_{D}l_{M}sin\left(\alpha_{2d}\right)} \end{array}$$ (38)

$$\begin{array}{c} l_{t}\text{=}\left(r_{d}\left(1\right)c_{\alpha_{\text{1}}d}+r_{d}\left(2\right)s_{\alpha_{1d}}-\rho_{o2}\left(1\right)-\rho_{o30}\left(3\right)s_{\alpha_{2d}}\right)/c_{\alpha_{2d}}-\rho_{o30}\left(1\right) \end{array}$$ (39)

where ***r***_*d*_(*i*) denotes the *i*-th element of the vector ***r***_*d*_. Defining the desired joint positions as ***q***_*td*_ = [*α*_1*d*_, *β*_2*d*_, *l*_*td*_], and considering the velocity compensator in Eq. (34), the hydraulic system model given by Eq. (25) can be transformed into the following form:


$$\begin{array}{c} {\dot{p}}_{L}\text{=}K_{\text{0}}f\left({\overset{―}{p}}_{L}\right)u-C_{tc}p_{L} \end{array}$$ (40)

To stabilize the system described by Eq. (35), the inner-loop controller is designed as:


$$\begin{array}{c} u_{c}=K_{pc}{\left(f\left({\overset{―}{p}}_{L}\right)\right)}^{-1}{\tilde{p}}_{L} \end{array}$$ (41)

where ***K***_*pc*_ is the controller gain, and a positive-definite diagonal matrix, ${\tilde{p}}_{L}$ = ***p***_*Ld*_ − ***p***_*L*_, and ***p***_*Ld*_ is generated by the outer-loop controller. To address the disturbance forces induced by ship motions, the outer-loop controller is formulated as:


$$\begin{array}{c} p_{Ld}\text{=}\overset{-1}{\underset{t}{D}}\left(-G_{t}+M_{m}{\ddot{q}}_{m}+C_{m}{\dot{q}}_{m}+K_{p}\left(q_{td}-q_{t}\right)+K_{I}\int \left(q_{td}-q_{t}\right)dt\right) \end{array}$$ (42)

Here, ***K***_*P*_ and ***K***_*I*_ are the controller gain matrices and also positive definite diagonal matrices. Consequently, the inner-loop controller can be rearranged into the following form:


$$\begin{array}{c} u={\left(K_{\text{0}}f\left({\overset{―}{p}}_{L}\right)\right)}^{\text{-}1}K_{pc}{\tilde{p}}_{L}+A_{\text{1}}{\left(K_{\text{0}}f\left({\overset{―}{p}}_{L}\right)\right)}^{-1}{\dot{q}}_{tm} \end{array}$$ (43)

**Fig 6:**
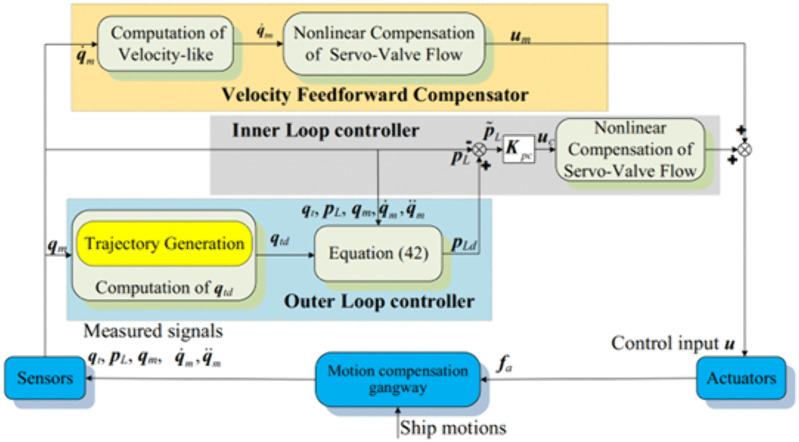
Block diagram of the modified control method.

The corresponding control block diagram is shown in Fig 6.

## Simulation results

In this section, a scaled model simulation is conducted to validate the effectiveness of the proposed control strategy. The simulation model is built using MATLAB/Simulink, with the assumption that ship motions can be perfectly measured. Other parameters of the motion-compensated gangway are listed in Table 1.

**Table 1:** Simulation parameters of the motion compensated gangway.

Parameters	Value	Parameters	Value
*m*_*TD*_	10000 kg	*L*_*M*_	1.0 m
***I***_*TDC*_	diag([1300;700;800]) kgm^2^	*p*_*s*_	28MPa
***ρ***_*o*1_ ***ρ***_TD_	(0.0, 0, −1.3) ^T^ m (−0.6, 0, 0.5)^T^ m	*D*_*m*_ *A*_1_	400ml/r 0.0245m^2^
*m*_*MB*_	2000 kg	*A*_t_	0.0118 m^2^
***I***_*MBC*_	diag([108,3500;3500]) kgm^2^	***Q***_*N*_	diag(800, 640, 640) L/min
***ρ***_*o*2_ ***ρ***_*MB*_	(0.5, 0, 0) ^T^ m (2.5, 0, −0.3) ^T^ m	*V*_N_	10
*m*_*TB*_	1200 kg	*C*_*tc*_	2 × 10^–11^ m^3^/(s·Pa)
***I***_*TBC*_	diag([65,1300;1400]) kgm^2^	*β*_e_	690 MPa
***ρ***_*TB*_	(−1.8, 0, −0.2) ^T^m	*l*_0_	1.811m
*L*_*D*_	1.51 m		

The initial joint positions of the motion-compensated gangway are set to [6.7, 0, −1.8], and the final position of the gangway end-effector in the inertial frame is [6.9, 0, −2]. In this study, a comparative analysis of three control methods is performed:

(1) The control method in [22], i.e., the conventional kinematic control method employing a PID controller with pressure feedback. The control law is as follows:

$$\begin{array}{c} u_{pi}\text{=}K_{p}\left(q_{td}-q_{td}\right)+K_{I}\int \left(q_{td}-q_{td}\right)dt-K_{dp}p_{L} \end{array}$$ (82)

After sufficient tuning, the control parameters are set as follows: ***K***_*p*_ = diag{10, 100, 40}, ***K***_*I*_ = diag {0.6; 30; 24}, *K*_*dp*_ = diag{1 × 10^*−*10^, 1 × 10^*−*7^, 3 × 10^*−*7^}.

(2) Improved Controller

The controller parameters are set as

***K***_*p*_ = diag {40 × 10^6^; 5 × 10^9^; 8 × 10^8^},
***K***_*I*_= diag {5 × 10^7^; 5 × 10^9^; 3 × 10^6^; 1 × 10^8^},
***K***_*pc*_= diag {5 × 10^-11^; 5 × 10^-11^; 5 × 10^-10^}.

**Fig 7:**
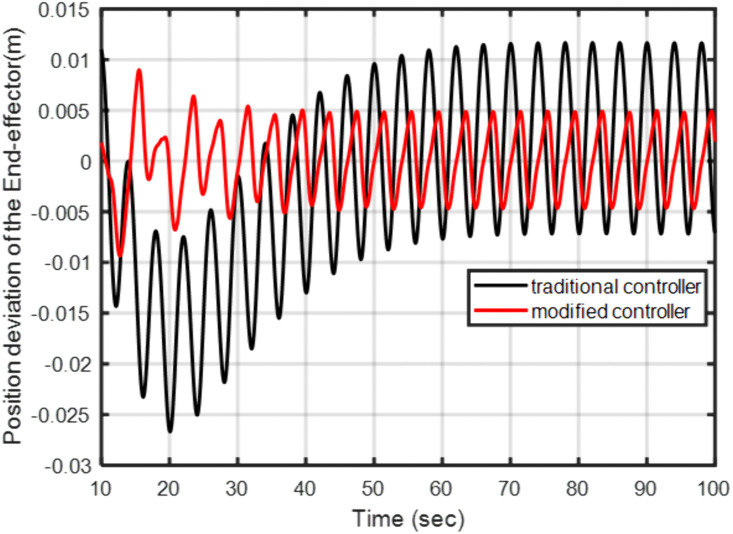
X-direction displacement deviation of ship sinusoidal motion in the Z-direction.

**Fig 8:**
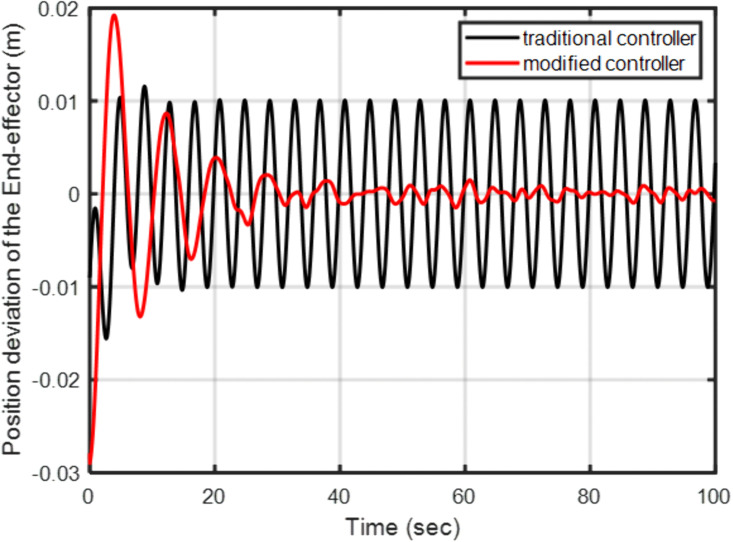
Y-direction displacement deviation of ship sinusoidal motion in the Z-direction.

**Fig 9:**
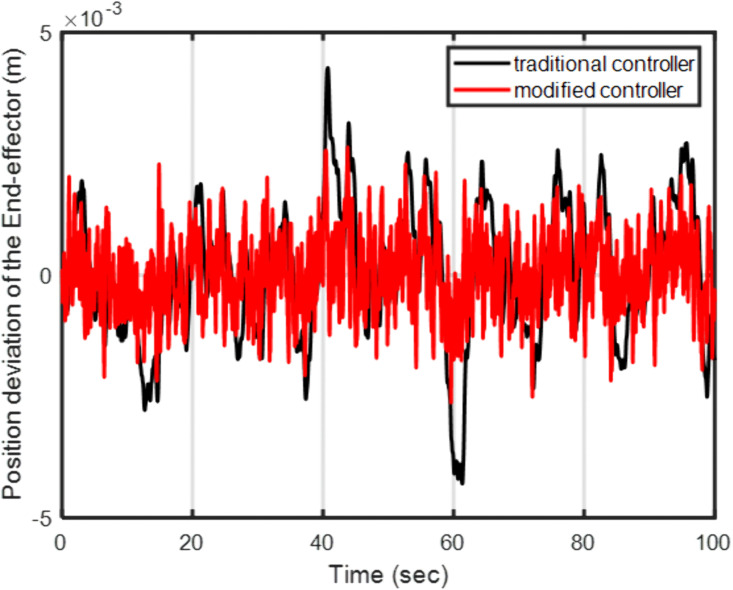
Z-direction displacement deviation of ship sinusoidal motion in the Z-direction.

The positional variation of the end-effector of the motion-compensated gangway in the inertial frame is utilized to evaluate control performance, which also represents the motion responses of the end-effector to ship motions. For all the simulation cases, the MRU sensor delay is set to 10 ms, and the white noise signals with a maximum amplitude and a frequency are 0.002 m (rad) and 2 Hz are utilized to simulate system noise, considering that the wave spectrum energy is primarily concentrated in the frequency range of 0.1–0.25 Hz. Single-degree-of-freedom simulation motions obtained from the motion sensor are set as follows: a sinusoidal signal with an amplitude of 0.21 m in the Z-direction at 0.25 Hz (Case 1), and a sinusoidal signal with an amplitude of 10 deg in the Rx-direction at 0.20 Hz (Case 2). The position deviation results of the end-effector are shown in Figs 7–9.

For the single-degree-of-freedom ship motion disturbance in the Z-direction, Figs 7–9 present the position deviations of the end-effector in the X-, Y-, and Z-directions in the inertial frame utilizing different controllers, respectively. After the system stabilizes, the conventional controller (black curve) results in maximum position deviations of 11.7 mm (X-direction), 10 mm (Y-direction), and 4.3 mm (Z-direction). In contrast, the improved controller (red curve) yields maximum position deviations of 4.9 mm (X-direction), 2 mm (Y-direction), and 3 mm (Z-direction). It can be seen that the improved controller enhances the overall control performance of the system.

**Fig 10:**
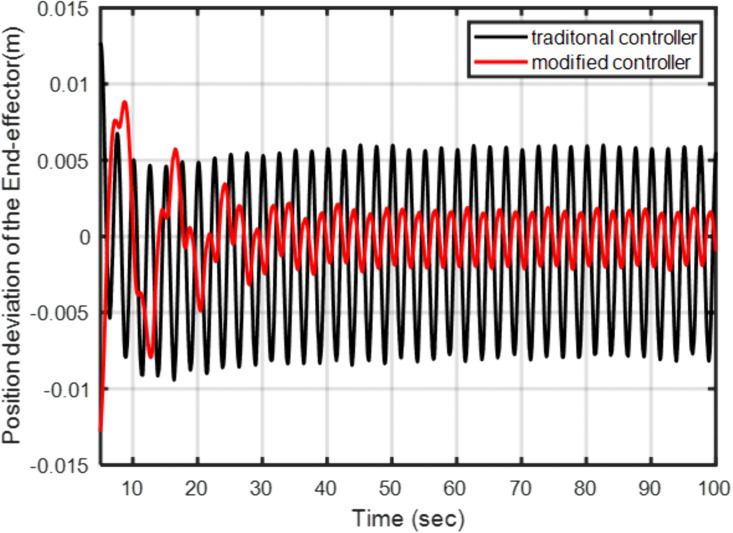
X-direction displacement deviation of ship sinusoidal roll motion about the X-axis.

**Fig 11:**
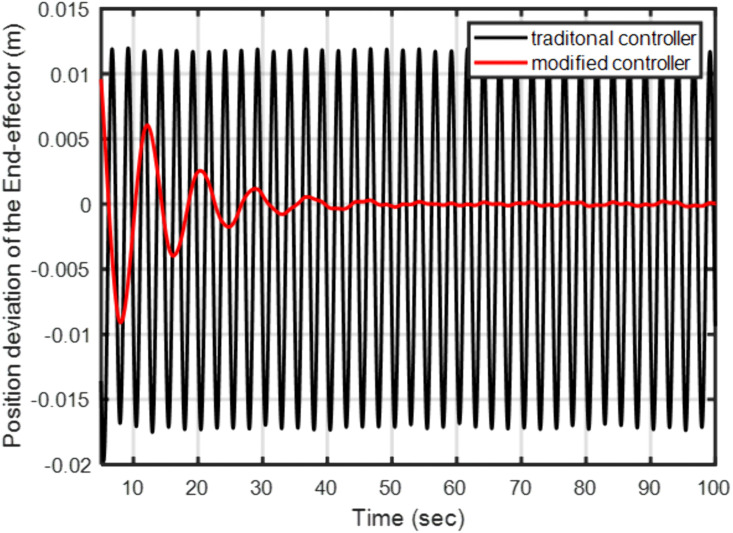
Y-direction displacement deviation of ship sinusoidal roll motion about the X-axis.

**Fig 12:**
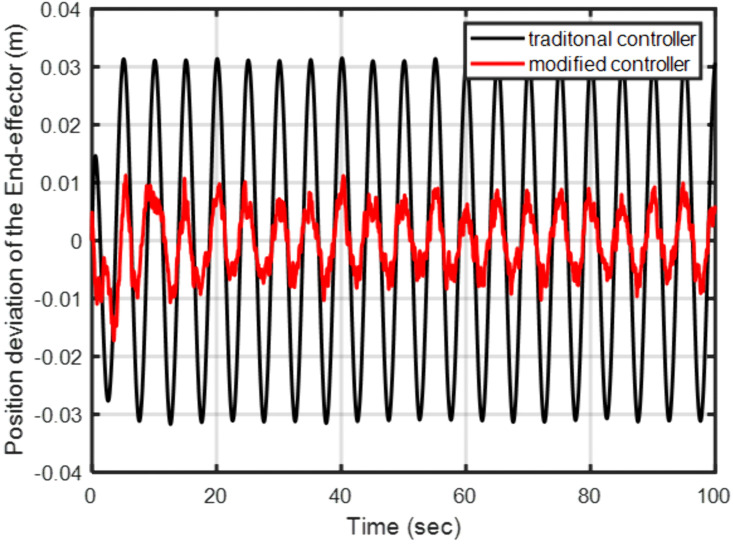
Z-direction displacement deviation of ship sinusoidal roll motion about the X-axis.

Figs 10–12 present the motion deviation of the gangway end-effector under ship roll (Rx-direction) motion disturbance. After the system stabilizes, the conventional controller yields maximum deviations of 5.9 mm (X-direction), 12 mm (Y-direction), and 31.4 mm (Z-direction). In comparison, the improved controller results in maximum position deviations of 2.0 mm (X-direction), 1 mm (Y-direction), and 11.2 mm (Z-direction). The improved controller demonstrates better overall performance.

**Fig 13:**
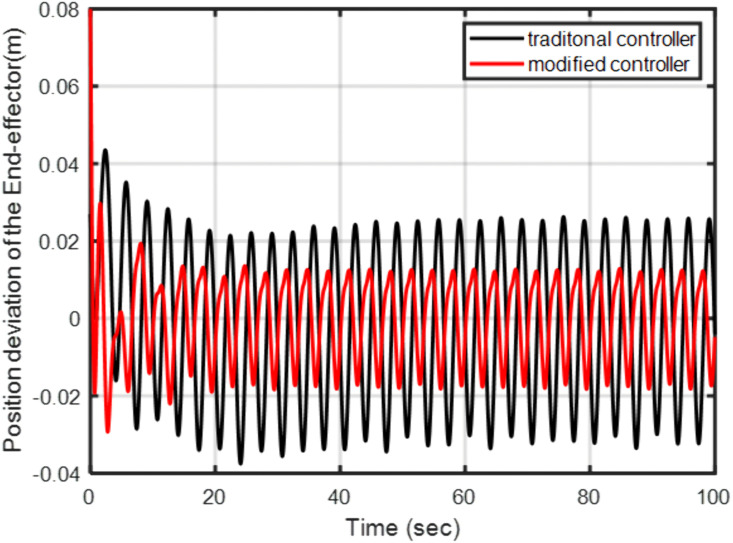
X-direction displacement deviation of three-degree-of-freedom ship motion.

**Fig 14:**
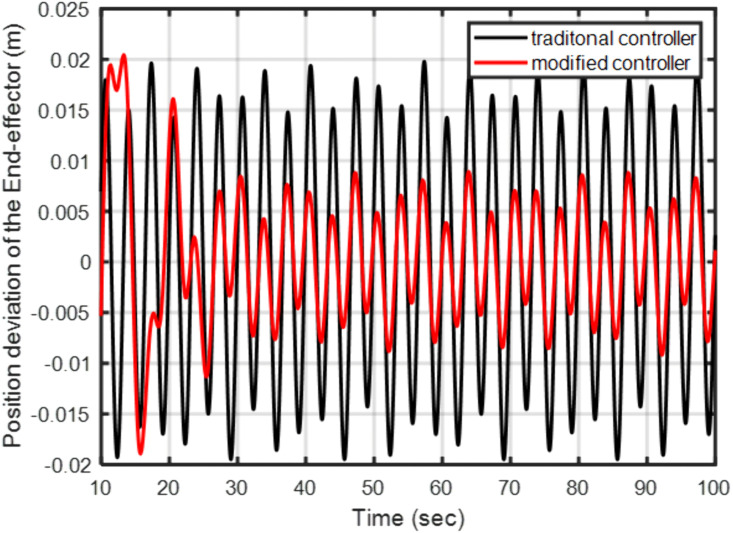
Y-direction displacement deviation of three-degree-of-freedom ship motion.

**Fig 15:**
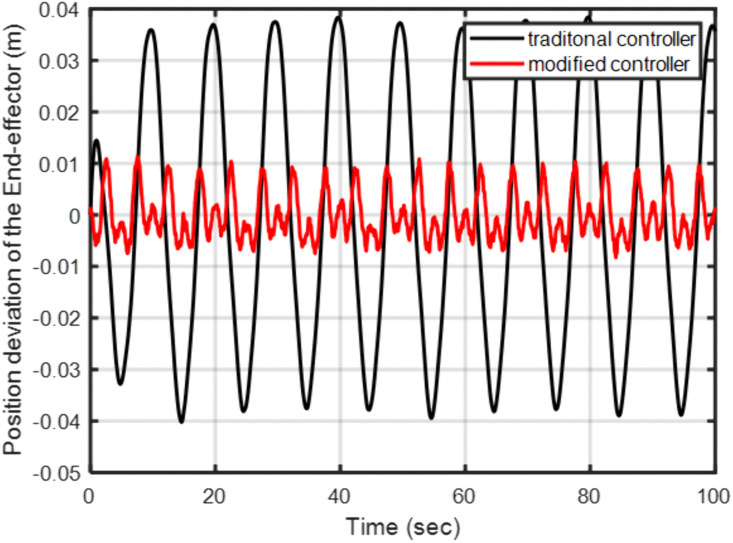
Z-direction displacement deviation of three-degree-of-freedom ship motion.

To provide a more comprehensive comparison of controller performance, a three-degree-of-freedom motion simulation is carried out, and the motions are defined as Case 3: *z*_*m*_ = 0.05sin(2π*t*/8), *ϕ*_*m*_ = 5sin(2π*t*/10), and *θ*_*m*_ = 3sin(2π*t*/5). The simulation results are shown in Figs 13–15.

Figs 13–15 show the motion deviation of the gangway end-effector under the compound three-degree-of-freedom ship motion disturbance in roll (Rx), pitch (Ry), and heave (Z). It can be observed that the conventional controller yields maximum end-effector motion deviations of 26.2 mm (X-direction), 19.3 mm (Y-direction), and 38.4 mm (Z-direction). n comparison, the improved controller results in maximum deviations of 17.1 mm (X-direction), 8.9 mm (Y-direction), and 10.3 mm (Z-direction).

**Fig 16:**
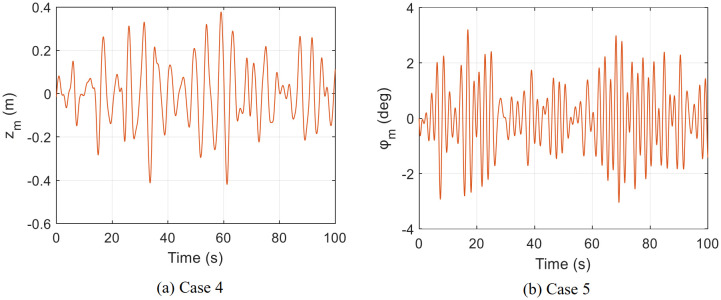
Random signal inputs (a) ship heave motion disturbance and (b) ship roll motion disturbance.

Furthermore, the Marine Systems Simulator (MSS) toolbox is used to generate random signals based on the JONSWAP wave spectrum (Fig 16). The parameters for the wave spectrum are set with a peak period of 5 s and a significant wave height of 0.6 m.

**Fig 17:**
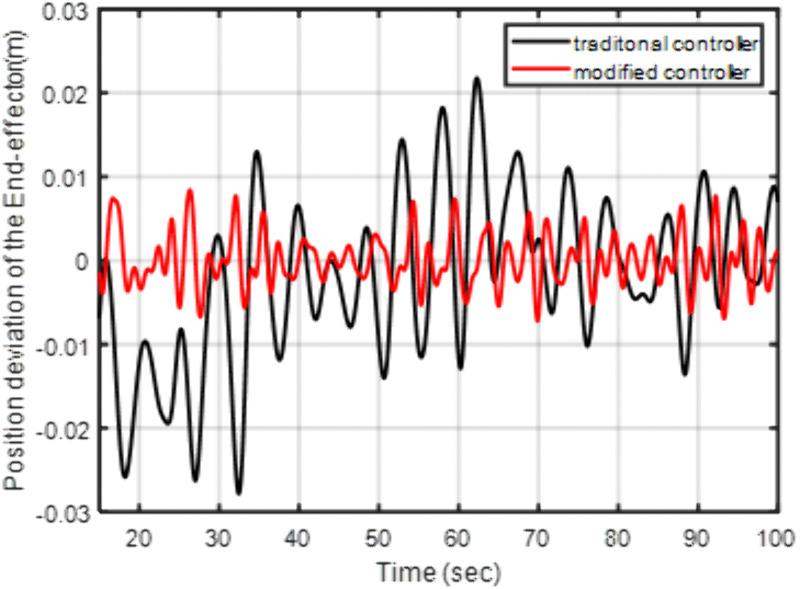
X-direction displacement deviation with ship heave motion disturbance.

**Fig 18:**
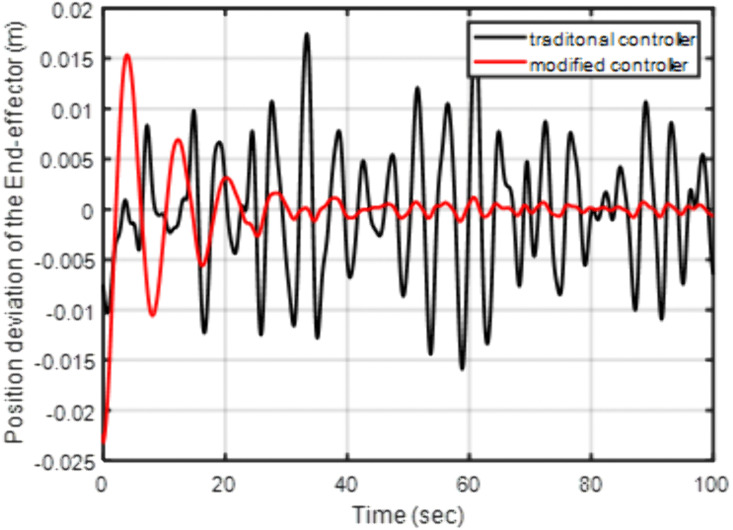
Y-direction displacement deviation with ship heave motion disturbance.

**Fig 19:**
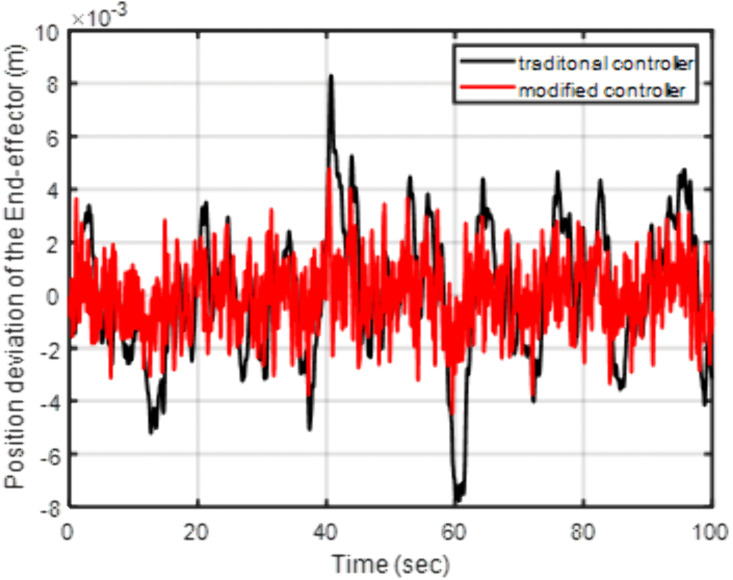
Z-direction displacement deviation with ship heave motion disturbance.

**Fig 20:**
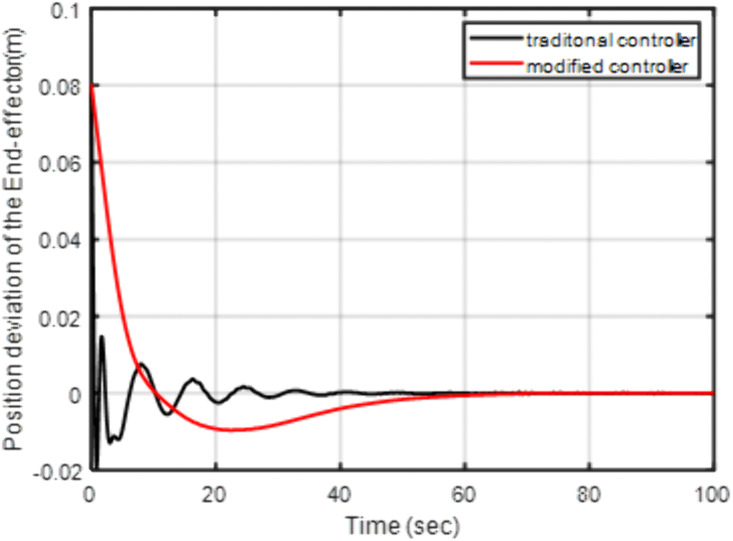
X-direction displacement deviation with ship roll motion disturbance.

**Fig 21:**
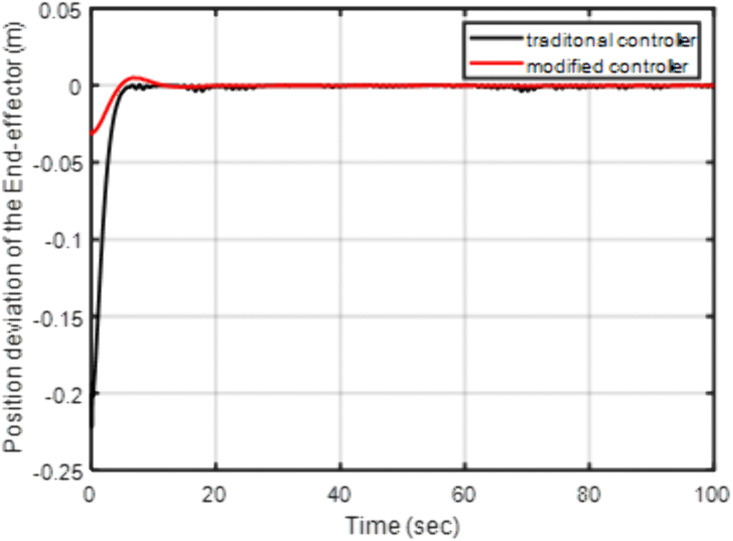
Y-direction displacement deviation with ship roll motion disturbance.

**Fig 22:**
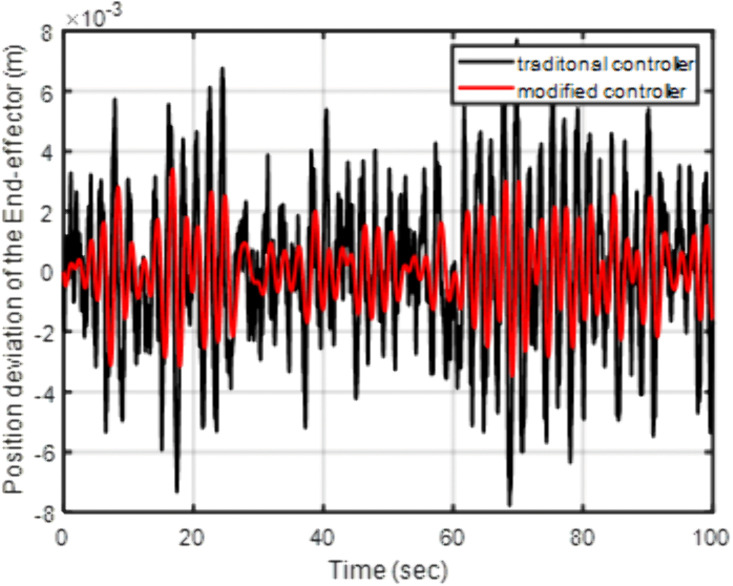
Z-direction displacement deviation with ship roll motion disturbance.

The simulation results for the ship heave (Z-direction) motion disturbance are plotted in Figs 17–19, the results indicate that the conventional controller yields maximum end-effector motion deviations of 21.8 mm (X-direction), 17.5 mm (Y-direction), and 8.3 mm (Z-direction), while the improved controller results in maximum deviations of 7.4 mm (X-direction), 1.2 mm (Y-direction), and 4.8 mm (Z-direction). The results for the ship roll (Rx-direction) motion disturbance can be seen in Figs 20–22, it can be seen that the conventional controller produces maximum deviations of 0 mm (X-direction), 0 mm (Y-direction), and 7.8 mm (Z-direction), whereas the improved controller achieves maximum deviations of 0 mm (X-direction), 0 mm (Y-direction), and 3.5 mm (Z-direction). Then all the simulation results are listed in Table 2.

**Table 2:** Summary of all the simulation results.

Simulation cases	X-direction (mm)	Y-direction (mm)	Z-direction (mm)
Case 1: traditional controller Case 1: modified controller	11.7 4.9	10 2	4.3 3
Case 2: traditional controller Case 2: modified controller	5.9 2	12 1	31.4 11.2
Case 3: traditional controller Case 3: modified controller	26.2 17.1	19.3 8.9	38.4 10.3
Case 4: traditional controller Case 4: modified controller	21.8 7.4	17.5 1.2	8.3 4.8
Case 5: traditional controller Case 5: modified controller	0 0	0 0	7.8 3.5

## Conclusion

This paper conducts an in-depth investigation into the dynamic model of a motion-compensated gangway system based on Kane’s method. Utilizing the derived model, the motion coupling between the gangway end-effector and ship motions is analyzed. Subsequently, an improved controller based on the multi-degree-of-freedom velocity compensator is designed to address the disturbance forces induced by ship motions. The results from the five simulation cases demonstrate that the control performance has been enhanced by more than 50%.

## Supporting information

S1 Dataset Dataset.ZIP archive containing all minimal anonymized simulation data required to reproduce all results and figures in this work. (ZIP)
